# The Historical Baseline of Hard Tick Records in Spain (1985–2024)

**DOI:** 10.3390/pathogens14020173

**Published:** 2025-02-09

**Authors:** Agustín Estrada-Peña, Miguel A. Habela Martínez-Estéllez, Carlos Pradera, Joaquim Castellà

**Affiliations:** 1 Department of Animal Health, Faculty of Veterinary Medicine, 50013 Zaragoza, Spain; 2 Instituto Agrolimentario de Aragón, IA2, 50013 Zaragoza, Spain; 3 Ministry of Human Health, 28014 Madrid, Spain; 4 Department of Animal Health, Parasitology and Parasitic Diseases, Faculty of Veterinary Medicine, 10003 Cáceres, Spain; mahabela@unex.es; 5 Anticimex 3D Sanidad Ambiental SA, 08174 Sant Cugat del Vallès, Spain; carlos.pradera@anticimex.es; 6 Department of Animal Health and Anatomy, Faculty of Veterinary Medicine, Autonomous University of Barcelona, 08193 Bellaterra, Spain; joaquim.castella@uab.cat

**Keywords:** ticks, Ixodidae, Spain, distribution, years 1985–2024

## Abstract

Ticks are important vectors of pathogens, producing diseases in animals and humans. The planning of information campaigns or prevention programs is heavily based on the knowledge of highly detailed data on tick distribution. This study reports unpublished data on the distribution of more than 30,000 tick specimens, collected by active surveys in the years 1985–2024 in Spain, from 2285 surveys in 1636 unique sites, providing coordinates with variable accuracy. The report covers new records of *Dermacentor marginatus*, *Dermacentor reticulatus*, *Haemaphysalis punctata*, *Haemaphysalis sulcata*, *Hyalomma marginatum*, *Hyalomma lusitanicum*, *Ixodes ricinus*, *Rhipicephalus bursa*, *Rhipicephalus hibericus*, and *Rhipicephalus sanguineus* (either s.s. or s.l.). Other species were sporadically collected. Many specimens have been re-examined for accurate identification according to current taxonomic views, mainly in the genus *Rhipicephalus*. We summarized this newly available information using the Köppen–Geiger climate classification. This compilation of unpublished tick records pinpoints the importance of the systematic monitoring of ticks. It is intended as the baseline over which the ongoing national tick collection program is built in order to track the long-term changes of tick distribution in Spain, because of the land use changes, the spread of invasive vertebrates, or the climate trends.

## 1. Introduction

Mapping the known distribution of ticks within a country or larger administrative divisions (e.g., a continent or a natural vegetation zone) is an ongoing effort essential for developing preventive measures for the public and evaluating control strategies for tick parasites affecting livestock [1,2,3]. It is, however, crucial that records are adequately georeferenced, as modelling efforts become meaningless if the original records lack the necessary spatial accuracy [4,5]. Official organisations such as the European Centre for Disease Prevention and Control (ECDC) in Europe routinely follow a standard practice, employing the NUTS3 aggregation scheme for tick mapping (see, e.g., https://www.ecdc.europa.eu/en/disease-vectors/surveillance-and-disease-data/tick-maps, accessed on 18 August 2024). Similarly, the United States Centers for Disease Control and Prevention (CDC) utilises county boundaries for tick mapping (see https://www.cdc.gov/ticks/data-research/facts-stats/geographic-distribution-of-tickborne-disease-cases.html, accessed on 18 August 2024). In both cases, the primary objective is to compile as much information as possible to inform the public.

Although such compilations are valuable resources for broad-scale studies on ticks, they typically lack references to hosts and precise coordinate data. These attributes are crucial for assessing the impact of ticks on human and animal health, enabling unambiguous mapping for further ecological research (see, e.g., https://vectormap.si.edu, accessed on 2 June 2024). Recent updates on tick fauna in various countries have increasingly incorporated coordinate data from tick collections [6,7,8], even for local-scale surveys [9] or in the preparation of digital maps (e.g., [10,11]). However, other compilations, such as those covering the entire Mediterranean region, have only reported the names of collection localities, making site recognition challenging (e.g., [12]).

The history of tick research in Spain is fragmentary. A compilation of the known tick species in Europe [13] included records of ticks in Spain, represented as points on a map; however, this was intended for informational purposes only and did not provide additional details regarding species or records under similar ecological conditions. For many studies, machine-readable coordinates are a fundamental requirement, yet such data cannot be reliably inferred from sketched maps. A recent study attempted a more comprehensive compilation of tick records, integrating machine-readable coordinate data into a geographical information system [14]. However, information on the date of collection and host species remained absent. In recent years, surveys have been published on ticks in Spain, offering a greater level of detail and expanding the national tick catalogue [15,16,17,18,19,20].

This study aims to update the known distribution of ticks in Spain, focusing on records of the family Ixodidae collected between 1985 and 2023. We summarise data on ixodid ticks collected by the co-authors of this study, which have not been published previously. Thus, this is not a bibliographic review but rather an update of tick species known to exist in Spain. The study also provides an overview of the climatic conditions under which the species were collected. Our objective is to establish a baseline of tick records that, together with existing published reports, will serve as a starting point for ongoing national surveys contributing to the National Plan against Ticks, coordinated by the Ministry of Health and the Ministry of Agriculture of Spain. This effort aims to provide researchers with an updated distribution of the most significant tick species in Spain, with explicit references to host species, collection dates, and geographic coordinates.

## 2. Materials and Methods

The new Ixodidae records included in this study comprise 1636 unique collection sites. As some sites were surveyed multiple times, the total number of tick collection events is 2285. For each record, the species, host, month of collection, and geographic coordinates are provided. A small number of Argasidae specimens were collected during the study period, primarily from pigeon nests in urban areas; these are not included in this compilation. Approximately 80% of records of Ixodidae were obtained from ticks feeding on hosts, while the remaining 20% were collected using flagging or dragging methods by the co-authors or members of their teams.

All records in the dataset are georeferenced. Approximately half of them were georeferenced in the field at the time of collection using various GPS devices. Older records (collected before approximately the year 2000) were not originally digitally georeferenced due to the unavailability of appropriate digital resources at the time. These records were initially mapped using UTM coordinates on high-resolution military maps, employing a compass, a ruler, and direct field knowledge. In 2022 and 2023, these records were reviewed and, where necessary, corrected using digital versions of the same maps provided by the Spanish National Geographic Institute and on-screen georeferencing tools (http://www.ign.es/web/ign/portal, accessed on 12 October 2024). In some cases, high-resolution, accurately georeferenced LANDSAT imagery (15 m per pixel) was used to compare field logs and original maps, ensuring the most precise possible georeferencing.

Approximately 30% of the specimens were obtained from hunting activities and submitted to our laboratories by various collaborators. In most cases, the only available location reference was the smallest administrative unit near the hunting site (e.g., a village). These specimens were georeferenced using online digital maps, obtaining on-screen coordinates corresponding to the centroid of the site. We estimated that the average positional error for records based solely on the neighbouring territory of a village is approximately 5 km. However, when a specific farm name was provided, the error was reduced to 20–50 m. Supplementary Table S1 contains the complete list of records and coordinates, along with an additional column indicating the assumed accuracy of each record, following an established procedure [6]. High accuracy (“h”, ±30 m) was assigned to coordinates provided in decimal degrees with at least four to five significant decimal places. Medium accuracy (“m”, ±1 km) was assigned to coordinates with two to three significant decimal places, as well as those from ticks collected from animals or humans. Low accuracy (“l”) was attributed to records referencing only the nearest village without further collection details. The category “unknown” (“u”) was used for ticks collected within large administrative divisions (e.g., NUTS2) where only the main town name was available as a reference; these account for only nine out of 1636 collection points (0.5%).

Most ticks collected from hosts were obtained from livestock, pets, or wild fauna. Collections from small mammals (e.g., Rodentia, Eulipotyphla) or birds have been published in part elsewhere [21,22]. Consequently, the host data in this study are skewed, and summarizing vertebrate hosts would present a biased perspective. Nonetheless, all host information is included in Supplementary Table S1 and is summarized in the results section. We recorded every collection on hosts and summarized as the percent of ticks of each species collected on vertebrates; we included the information tabulated for the genera of vertebrates (not species) to avoid overdispersion of records that lack ecological or phylogenetic information.

Another key aspect of the dataset is the date of collection. Some records lack specific information on the day or even the week of collection. The decision to report the month rather than an exact date (day, month, year) was made to aggregate data while maintaining coherence in reporting. However, it is important to note that most records were obtained through opportunistic sampling rather than surveys planned for specific times and locations. For each species and site, we include a series of numbers (from 1 to 12) indicating the months in which the species was collected at that site. This is intended to complement the information on the phenology of certain species has already been published [22,23,24].

**Figure 1:**
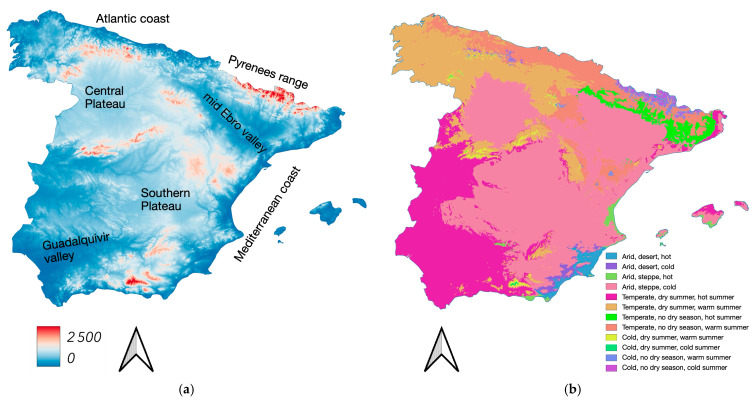
(**a**) A Digital elevation model (DEM) of continental Spain (without the Canary Islands) ranging from the sea level to 2500 m a.s.l. Highest elevation in the territory is 3404 m; values above 2500 m to the top limit have been included in the top category to better accommodate the range of colours. Resolution is 250 m. The map also includes the denominations of largest geographical regions (not administrative areas) that are referred to in the text. (**b**) A map of continental Spain (without the Canary Islands) of the Köppen–Geiger classification over the territory, obtained from [21]. Resolution is 1 km.

We have attempted to summarize the tick distribution data within a coherent environmental framework based on climate and vegetation. However, the spatial accuracy of some records is insufficient for direct comparison with high-resolution explanatory variables, such as those in the harmonized “Coordination of Information on the Environment” (CORINE) dataset. CORINE classifies European land cover into 44 categories with a spatial resolution of 100 m, which is finer than the resolution of many records georeferenced using on-screen coordinates. Consequently, a considerable number of records might be incorrectly assigned to vegetation categories. To mitigate this issue, we used a recent update of the Köppen–Geiger climate classification [25,26]. This classification divides global land areas into climate zones based on threshold values and the seasonality of monthly air temperature and precipitation (see [26] for a comprehensive explanation and an overview of the resulting maps). The Köppen–Geiger system has been widely used for tick distribution studies [27,28,29]. Basic details on the geographical and climatic context of the collections are presented in Figure 1.

We used a heat map for representation of the categories of the Köppen–Geiger classification to which ticks are associated. Since every record is geo-referenced in accordance with the resolution of the descriptive layer [26], we calculated the percent of records of each tick species as located in the Köppen–Geiger categories. We then transformed the table of tick records/Köppen–Geiger categories to the heatmap, in which colours mean the percent of times a tick species has been collected in such a category. We applied a k-means method (using internal routines in the “Orange” programming environment (https://orangedatamining.com/, last updated 14 October 2024)). The k-means produced a clustering of the species of ticks according to the similarity of the climate features at the collection sites yielding a visual comparison of the climate preferences.

We report newly records for *Dermacentor marginatus*, *Dermacentor reticulatus*, *Haemaphysalis punctata*, *Haemaphysalis sulcata*, *Hyalomma marginatum*, *Hyalomma lusitanicum*, *Ixodes ricinus*, *Ixodes frontalis*, *Ixodes ventalloi*, *Rhipicephalus bursa*, *Rhipicephalus hibericus*, and *Rhipicephalus sanguineus* (either sensu stricto or sensu lato). Maps have been prepared for these commonly collected species, along with a summary of the climatic regions in which they were recorded. Additionally, we provide brief comments on rarely collected species, some of which were represented by only a single specimen in 37 years of active and passive surveillance. Every detail for records of poorly collected species is included in the Supplementary Table S1. The information in the supplementary data has been included in a machine-readable format.

## 3. Results and Discussion

**Figure 2:**
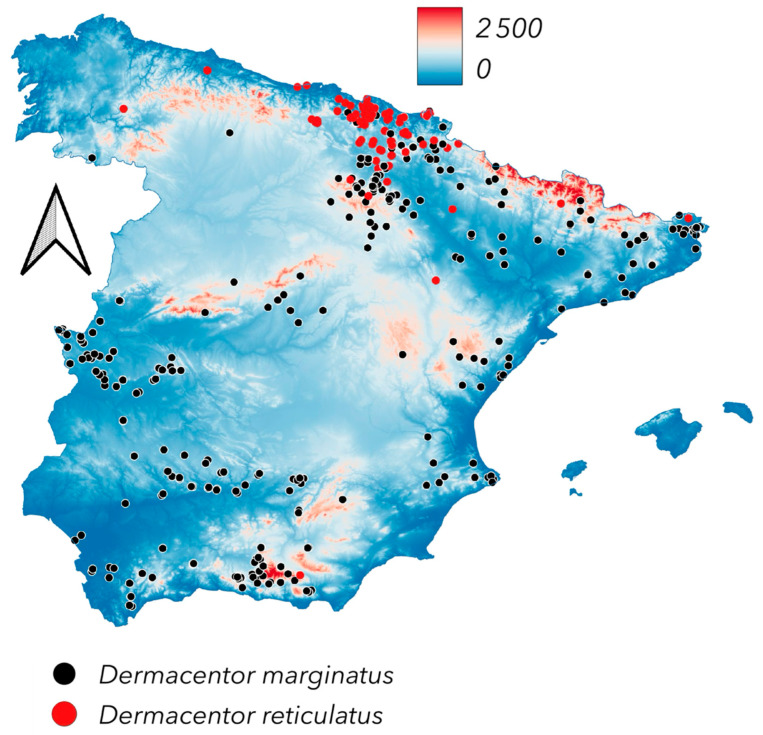
The newly reported distribution of *Dermacentor marginatus* and *Dermacentor reticulatus*.

Figure 2 shows the distribution of *D. marginatus* and *D. reticulatus* in the target territory. These species exhibit a disjoint distribution, with an area of overlapping in northern Spain, characterized by low-elevation hills, high humidity, and relatively mild temperatures. The highest elevations in this area may experience cold and rainy winters. In this region, *D. reticulatus* is restricted to the northern coastal portion, while *D. marginatus* extends into the drier southern areas. Both species are widespread in Europe and the British Isles [6,7,10,12]. A detailed study on their co-distribution across parts of Europe has been published [28], supporting the pattern observed in Spain. *Dermacentor marginatus* is widely distributed in warm areas of Spain and appears to be associated with herbivores, based on adult collections. It follows valleys and river systems, reaching medium-high elevations of up to 1000 m a.s.l., but is absent at higher altitudes.

**Figure 3:**
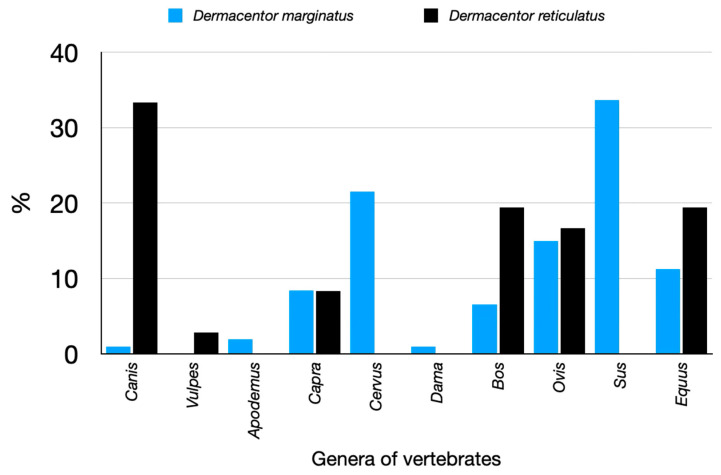
The recorded hosts for *Dermacentor marginatus* or *Dermacentor reticulatus*, summarized as the percent of collections on genera of vertebrates.

Both species of *Dermacentor* have different hosts. The adults of *Dermacentor reticulatus* can be collected on both Carnivora and Artiodactyla/Perissodactyla, while *D. marginatus* seems to be present only on ungulates (Figure 3).

**Figure 4:**
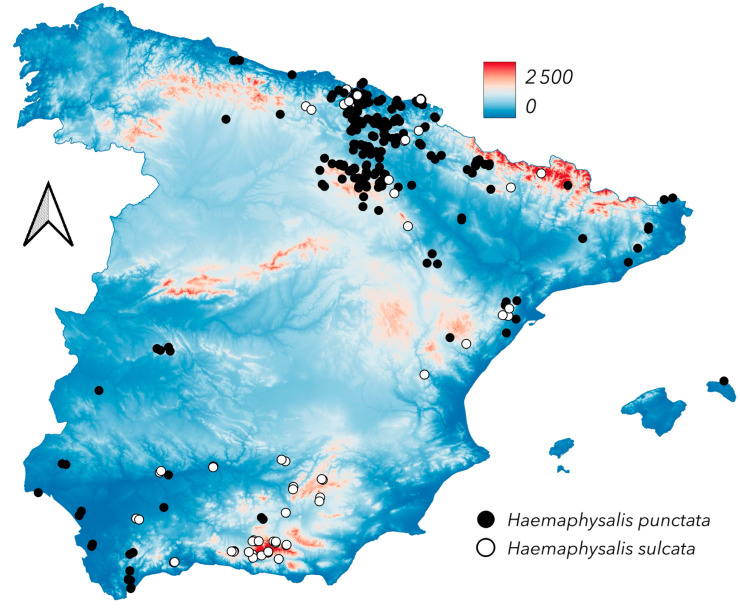
The newly reported distribution of *Haemaphysalis punctata* and *Haemaphysalis sulcata*.

**Figure 5:**
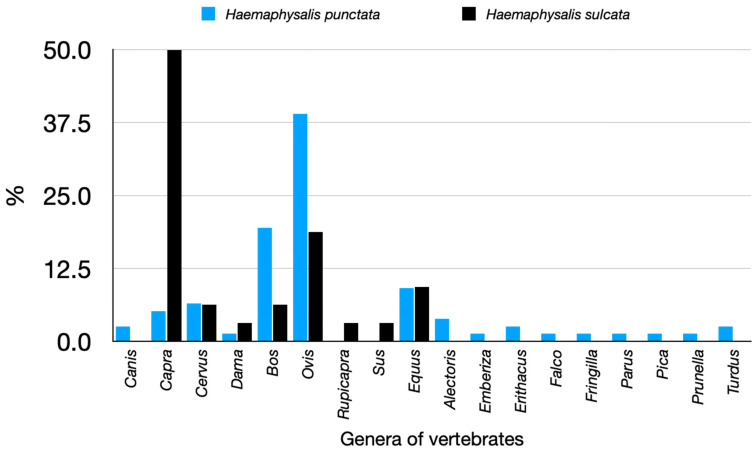
The recorded hosts for *Haemaphysalis punctata* or *Haemaphysalis sulcata*, summarized as the percent of collections on genera of vertebrates.

Figure 4 presents the updated records of the genus *Haemaphysalis* in Spain. *Haemaphysalis punctata* is a well-known species primarily found in northern Spain, commonly occurring in wet areas and at moderate elevation, such as the Pyrenees, near the Spain–France border, at approximately 1200 m a.s.l. However, it is also present in a few southern Spanish localities. In contrast, *H. sulcata* is found in drier environments, at low to medium altitudes, and is rare in humid regions. Several records from southeastern Spain, associated with mid-elevation hills along the Guadalquivir River valley (see Figure 1), suggest that its distribution may be influenced either by local climatic factors or by the distribution of its hosts, which are restricted to areas with suitable resources. Both species are known from most European countries, except those in the north [30,31]. *Haemaphysalis sulcata* has more xerophilous preferences and is well represented in Mediterranean Europe, North Africa [13,31], and extensive areas of Central Asia [32,33,34], reaching as far as China [35]. The majority of *H. punctata* nymphs reported in this study were obtained from birds, complementing previous reports [21]. In contrast, *H. sulcata* immatures were frequently found on lacertid lizards, in line with earlier studies [36,37]. Figure 5 summarizes the findings of these two species of *Haemaphysalis* on different hosts. Additionally, we report the first finding of *Haemaphysalis erinacei* nymphs in the mid-Ebro River valley and confirm permanent populations of *Alloceraea inermis* (previously in the genus *Haemaphysalis*) along the northern Atlantic coast. The records of the latter two species are not mapped in Figure 4.

**Figure 6:**
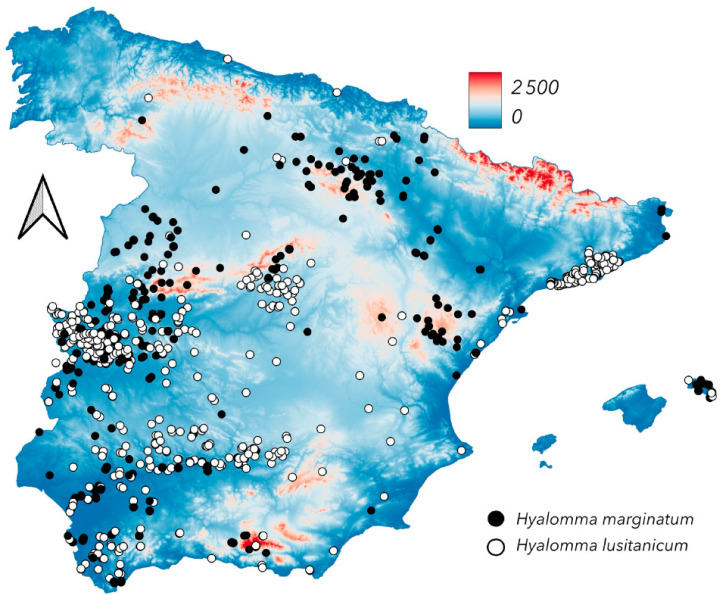
The newly reported distribution of *Hyalomma marginatum* and *Hyalomma lusitanicum*.

**Figure 7:**
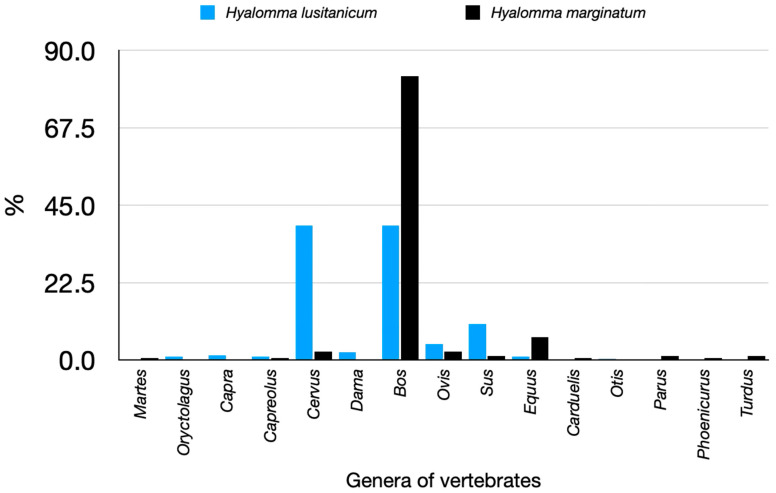
The recorded hosts for *Hyalomma lusitanicum* or *Hyalomma marginatum*, summarized as the percent of collections on genera of vertebrates.

The distribution of *H. lusitanicum* appears to follow major river systems and is likely influenced by the presence of rabbits and large ungulates (both wild and domestic) (Figure 6 and Figure 7). This pattern is particularly evident in southwestern Spain, where most *H. lusitanicum* collections are concentrated along the Guadalquivir River. This is the first historical map and georeferenced dataset of *H. lusitanicum* in Spain, a species of interest due to its potential role in the transmission of Crimean–Congo haemorrhagic fever virus (CCHFV) or *Orthonairovirus hemorragiae*, a deadly disease affecting humans [38]. The virus has been reported across the Mediterranean, the Near East, the Middle East, and as far as China, as well as in numerous African countries. Several tick species, acting as vectors and reservoirs, and various vertebrate hosts, sustaining tick populations and facilitating viral circulation, are involved in CCHFV transmission globally. However, no laboratory studies have confirmed that *H. lusitanicum* can transmit the virus. The first detection of CCHFV in Spain was from *H. lusitanicum* feeding on *Cervus elaphus* [39], with subsequent surveys detecting viral RNA in this tick species [40,41]. However, the presence of viral RNA in these ticks could result from infected host blood rather than active viral transmission, introducing a potential bias in assessing their public health importance. The full range of *H. lusitanicum* is restricted to Spain and nearby Mediterranean countries, including Portugal, Corsica, Sicily, Morocco, Algeria, and Tunisia [https://www.ecdc.europa.eu/en/disease-vectors/surveillance-and-disease-data/tick-maps, accessed on 1 August 2024]. Its abundance in suburban areas can reach concerning levels, particularly where human settlements overlap with high densities of rabbits and wild ungulates (notably wild boar, *Sus scrofa*, and red deer, *Cervus elaphus*).

Conversely, *H. marginatum* is widespread across Spain, occupying large areas of dry, warm environments. It is absent from the northernmost regions, which are colder and wetter, and from high mountains above 1200 m a.s.l. However, it occurs in medium-elevation hills of the pre-Pyrenean range (northeastern records in Figure 2). *Hyalomma marginatum* is the principal CCHFV vector within its range [https://www.ecdc.europa.eu/en/disease-vectors/surveillance-and-disease-data/tick-maps, accessed on 1 August 2024], though the epidemiological situation in Spain remains unclear, as only a few human cases have been linked to identified tick bites. A partial review of *Hyalomma marginatum* hosts in Europe has been published [42].

Limited data exist on the ecological factors defining the observed distributions of *H. marginatum* and *H. lusitanicum* in Spain and other European countries over extended periods. A comparative ecological study on both species in Spain has been published [43], though the climatic determinants of their long-term persistence remain poorly understood. Several studies have examined climate influences on *H. marginatum* distribution [44,45,46], but field validation is still needed [47,48]. The concept of a threshold temperature for *H. marginatum* survival and reproduction, often used as an indicator of habitat suitability, may be an oversimplification, as multiple environmental factors likely interact to regulate tick physiology [49]. These interactions may also vary across spatial and temporal scales.

Other *Hyalomma* species collected in Spain over the past 35 years include *Hyalomma anatolicum*, *Hyalomma excavatum*, and *Hyalomma scupense*. While *H. scupense* has already been documented in Spain [40], our dataset contains too few records for further discussion.

Figure 7 summarizes the genera of vertebrates on which either *H. lusitanicum* or *H. marginatum* have been collected. Adults of both species are common on livestock and wild ungulates (almost 90% of collections of *H. marginatum* were performed on cattle).

**Figure 8:**
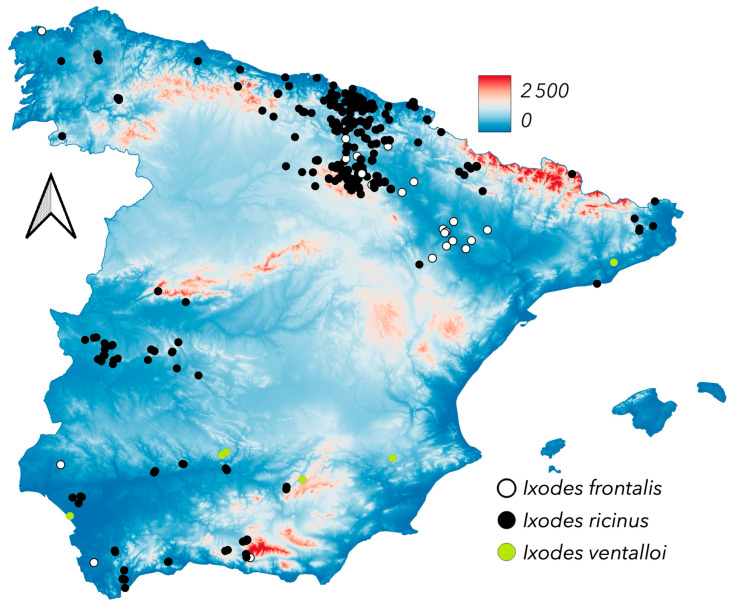
The newly reported distribution of *Ixodes frontalis*, *Ixodes ricinus*, and *Ixodes ventalloi*. Other species of the genus *Ixodes*, like *Ixodes acuminatus*, *Ixodes trianguliceps*, *Ixodes canisuga*, and *Ixodes hexagonus,* were collected in few places and are not mapped.

The genus *Ixodes* is probably the one with more representatives in Spain. We found some still unreported specimens of the bat-parasitizing ticks *Ixodes simplex* and *Ixodes vespertilionis* on either *Miniopterus* spp. or *Rhinolophus* spp. These specimens were collected in caves and are therefore not included in maps with coordinates to protect the bat colonies from occasional visitors. Furthermore, since they are cave associated ticks, it is not possible to allocate a climate category. Both *Ixodes canisuga* and *Ixodes hexagonus* were found on Carnivora (few records to map). These two species are common on wild carnivores, as reported for other sites in Europe [13,14,31,36]. *Ixodes ricinus* is one of the most collected *Ixodes* in Spain [15], because its prevalence in suitable habitats or because its importance as vector of multiple pathogens to humans [14,18,19,22]. Figure 8 shows that *I. ricinus* is restricted to the northern parts of the surveyed territory, where climate is colder and wet. It is also present in medium elevation hills (around 800 m a.s.l.) in which the heat in the summer is not extreme. *Ixodes ricinus* is present in a wide territory in Europe, from south-western, including Portugal and Spain, to northern countries and the European limit with western Russia [https://www.ecdc.europa.eu/en/disease-vectors/surveillance-and-disease-data/tick-maps, accessed on 1 August 2024]. The surveys that are being carried out in southern Spain (years 2024 to 2026) will confirm if the tick is still present in the south of the territory, because of the extreme changes produced by the climate trends, impacting a tick that is expected to be very sensitive to heat and dryness [50]. Records of *I. ricinus* in Spain point out a large variety of hosts, including Carnivora, small mammals, and ungulates. The apparent absence of *I. ricinus* on birds (as immatures, commonly reported across Europe [31]) is largely due to the concentration of bird surveys in parts of the Ebro mid valley (see Figure 1), which is largely unsuitable for *I. ricinus*.

**Figure 9:**
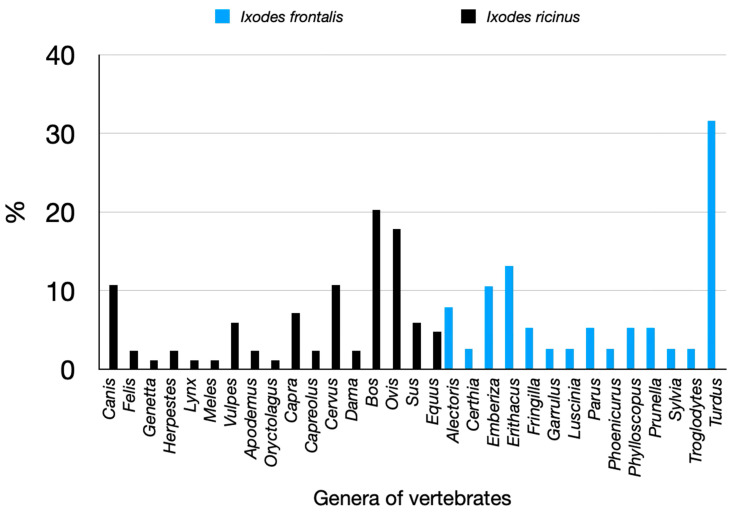
The recorded hosts for *Ixodes frontalis* or *Ixodes frontalis* and *Ixodes ricinus*, summarized as the percent of collections on genera of vertebrates. *Ixodes ventalloi* has been collected exclusively on Oryctolagus; the few collections of adults of *Ixodes hexagonus* and *Ixodes canisuga* were performed exclusively on Carnivora.

*Ixodes frontalis* is a common tick in Spain, associated to birds nesting in tree holes (see Figure 9). Most of the specimens have been collected while feeding on birds carrying out short local, non-migratory flyways. The coordinates of these records may not reflect the actual source of the tick. Last, but not least, *I. ventalloi* is a relatively uncommon species associated to the Mediterranean rabbit, *Oryctolagus cuniculus*. The tick has been collected in a few sites in Spain.

Ticks of the *Rhipicephalus* genus exhibit diverse distribution patterns in Spain. Recent taxonomic revisions have significantly altered our understanding of the complex *R. sanguineus* genus in Europe and North Africa, including the redescription of *R. sanguineus* [51], reinstatement of *Rhipicephalus rutilus* and *Rhipicephalus secundus* [52,53], and description of the new species *R. hibericus* [54]. In this group of species, only *R. hibericus*, *R. pusillus,* and *R. sanguineus* s.s. seem to exist in Spain (see [55] for a list of valid species of the *R. sanguineus* group at the time of writing).

Given these systematic changes, many specimens were re-examined before to be included in this study, though approximately 10% of collections had been used in prior studies and were unavailable for reassessment. As a result, some collections were reclassified as *R. hibericus*, while others were confirmed as *R. sanguineus* s.s. Unverified specimens have been designated *R. sanguineus* s.l.

**Figure 10:**
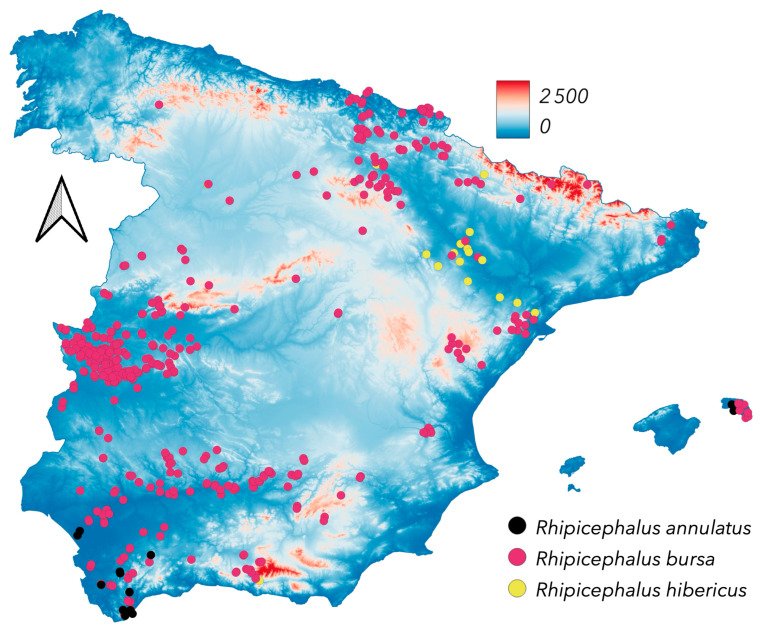
The newly reported distribution of *Rhipicephalus annulatus*, *Rhipicephalus bursa*, and *Rhipicephalus hibericus*.

**Figure 11:**
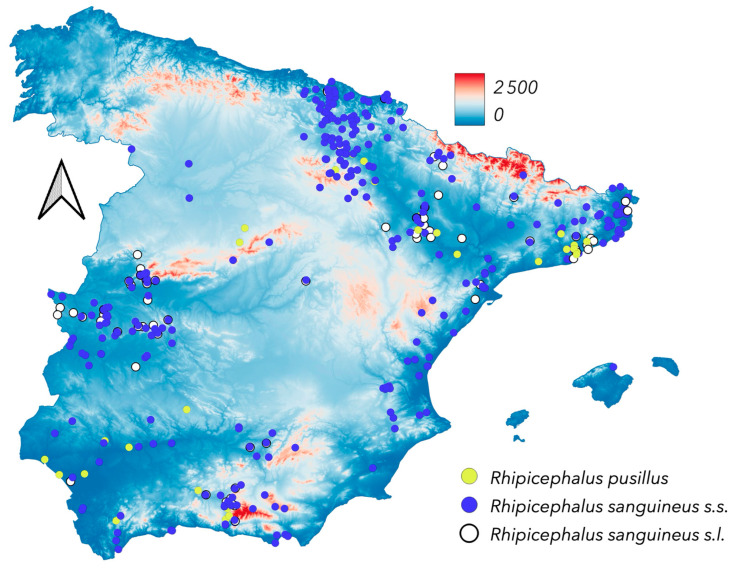
The newly reported distribution of *Rhipicephalus pusillus*, *Rhipicephalus sanguineus* s.s., and *Rhipicephalus sanguineus* s.l.

The most prevalent *Rhipicephalus* species in Spain is *R. bursa* (Figure 10), found in nearly all surveyed regions across diverse environmental conditions and host species (both domestic and wild ungulates). *Rhipicephalus annulatus* is rare in Spain, recorded only in the Balearic Islands, where it parasitises cattle under arid conditions; recent unpublished surveys found the species in southern Spain, but these records lack the locality of collection. The finding of *R. hibericus* feeding on wild carnivores underscores the need for additional morphological and molecular analyses. *Rhipicephalus sanguineus* s.s., an endophilous tick, is widespread but closely associated with dogs and human-made shelters such as kennels. Consequently, its mapped distribution (Figure 11) may not fully reflect its actual range. Adults of *R. sanguineus* s.s. have not been collected from ruminants, and re-examined specimens from such hosts were identified as *R. hibericus*, which is currently known from an extensive region in the mid-Ebro River valley (see Figure 1 and Figure 10). *R. pusillus*, which exclusively parasitizes the Mediterranean rabbit, *Oryctolagus cuniculus*, has been found a variety of sites in the surveyed territory. It is interesting to note that both *I. ventalloi* and *R. pusillus* are associated with *O. cuniculus*; however, *I. ventalloi* is rare in Spain, suggesting that other environmental features of the shelters of rabbits could indicate its suitable habitat. *Rhipicephalus pusillus* has been consistently documented in Spain [56,57,58,59,60].

**Figure 12:**
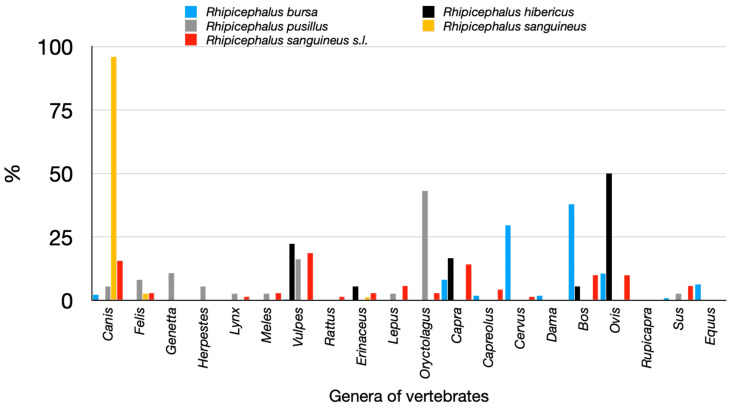
The recorded hosts for *Ixodes frontalis* and *Ixodes ricinus*, summarized as the percent of collections on genera of vertebrates.

Figure 12 shows the tabulation of the records of ticks of the genus *Rhipicephalus* collected while feeding on a large variety of hosts. Of interest, *R. sanguineus* s.s. has been found restricted to *Canis familiaris*; the records on *Vulpes* sp. were reidentified as *R. hibericus. Rhipicephalus pusillus* is associated with *O. cuniculus*, but it has been found on animals well known to predate on rabbits, like the genera *Genetta* and *Vulpes*. On the other hand, the widespread *R. bursa* is a common parasite of ruminants. Of note, the specimens of *R. sanguineus* probably belong to different taxa, because the wide range of hosts over which they have been collected. The re-examination of these specimens was not possible.

**Figure 13:**
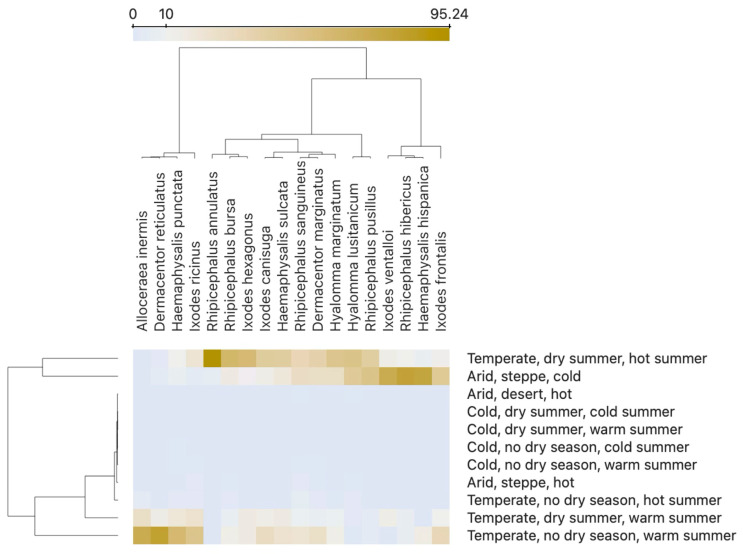
A heatmap representing the association of 18 species of ticks with climate categories according to the scheme Köppen–Geiger, in peninsular Spain, in the period 1985–2023. The colours in the chart and the colour gradient bar display the percent of times a given tick species was found to be associated with a climate category, using the coordinates for georeferencing. To enhance the contrast, colours are centred at the value of 10% (see the gradient bar, on top of the figure). Clustering was done using the k-means method. It revealed two large groups of ticks (top clustering), the second (right) further divided into two categories. All the categories where ticks were collected are displayed, but several of them (“cold”, “arid-desert”, and “temperature-hot summer”) yielded very few specimens (less than 0.5%).

Figure 13 shows the associations of the ticks collected to the Köppen–Geiger classification over the target territory. All the possible categories of the scheme existing in Spain were included in the figure. We observed a cluster of tick species that have been collected always in temperate areas, with warm summer, with or without dry season. Species in the cluster are *A. inermis* (not included in figures because only a few records were available), *D. reticulatus*, *H. punctata*, and *I. ricinus*. These four species represent the group of ticks that prefer cold environments in Spain that may be common only in humid regions and that “link” the tick faunal composition in the Iberian Peninsula to that of central Europe [13]. The rest of species are typical of a Mediterranean environment and are mainly separated by the features that delimit “temperate” and “arid” categories. Several species belonging to the genera *Dermacentor*, *Rhipicephalus,* and *Hyalomma* are present in both temperate and arid environments. Both *H. marginatum* and *H. lusitanicum* have been collected in temperate areas, with or without dry season, with warm or hot summer, but also are common arid conditions of cold steppe. One of the main characteristics of the ticks colonizing mediterranean habitats seems to be its high resilience to cold winters.

## 4. Conclusions

For decades, tick records in Spain have been accumulated in academic collections, or specimens have been utilized for other studies, such as essential research on pathogens of relevance to animal and human health or the development of regional-scale reports. As a large national project is currently underway to monitor changes in tick distribution across Spain, it is necessary to make available a comprehensive list of collections recorded between 1985 and 2023 as a baseline for future comparisons. The tick fauna of Spain reflects the anticipated mixture of Mediterranean species—adapted to warm or hot climates, often characterized by dry summers—and species associated with the tick fauna of Central Europe. The latter group is represented by *Ixodes ricinus*, *Dermacentor reticulatus*, and *Haemaphysalis punctata*, likely persisting as populations following the retreat of ice after the last glaciation. These species are commonly found in the northern regions of Spain, at mid-elevations, under a temperate climate without a dry season.

In addition to several endophilous species, ticks with Mediterranean-like ecological preferences include *Rhipicephalus bursa*, *Dermacentor marginatus*, *Hyalomma marginatum*, and *Hyalomma lusitanicum*. The two *Hyalomma* species exhibit partially overlapping distributions but differ in their preferences for temperate or colder areas with dry, hot summers. However, it is necessary to consider this study as a preliminary list of records, which has gaps in time and space. The surveys span 38 years and were not systematically carried out at defined moments of the year, and the collection pressure has not been similar in every part of the country.

This compilation, consisting of both active and passive surveillance and comprising approximately 30,000 specimens from 1636 collection sites, highlights the necessity of systematic monitoring of vector distribution changes to inform and develop effective pathogen prevention programs, or to detect changes in distribution patterns.

## Data Availability

All the data are included in this paper and its Supplementary Table S1.

## Supplementary Materials

The following supporting information can be downloaded at https://www.mdpi.com/article/10.3390/pathogens14020173/s1. Table S1: Complete list of tick records, including species, coordinates, administrative names and references, and date (months) of collection(s) at a given site.

## Abbreviations

The following abbreviations are used in this manuscript:

CCHF	Crimean–Congo haemorrhagic fever
DEM	digital elevation model
